# Optimizing the setup of multienvironmental hybrid wheat yield trials for boosting the selection capability

**DOI:** 10.1002/tpg2.20150

**Published:** 2021-09-19

**Authors:** Moritz Lell, Jochen Reif, Yusheng Zhao

**Affiliations:** ^1^ Leibniz Institute of Plant Genetics and Crop Plant Research (IPK) Seeland D‐06466 Germany

## Abstract

The accuracy of genomic prediction increases with increasing heritability, and thus the challenge of optimizing the design of multienvironment yield trials under a limited budget arises. With this in mind, we aimed to find the best of several options to sparsely distribute a fixed number of plots across different environments to increase the accuracy of hybrid performance prediction. We used a comprehensive published genomic and phenotypic data set of 1,604 winter wheat (*Triticum aestivum* L.) hybrids and compared several commonly used biometric models for phenotypic data analysis in a resampling study to identify the one that most accurately estimated the hybrid performance in different imbalanced trials. Our results showed that when using information about genotypic relationships, genotypic values were more strongly associated with the reference values than when this information was ignored. In addition, a balanced environmental sampling resulted in an adequate characterization of each environment and increased the accuracy for estimating the hybrid performance. One promising design involved dividing the genotypes into equally sized subgroups that were tested in a subset of environments, with the constraint that the subgroups overlapped with respect to the environments. This scenario appears to be particularly appropriate, as it provided both high accuracies in the estimates of genotypic values and had low variability resulting from the data sample used. Thus, we were able to clearly demonstrate the utility for optimizing the design of multienvironment hybrid wheat yield trials in times of genomic selection.

## Core Ideas

- In preliminary trials, evaluating candidates sparsely in more environments improves accuracy.
- A prerequisite for that are biometric models that consider candidate relatedness.
- Joint analysis of concurrent trials can improve accuracy without logistic changes.

**Abbreviations**
BLUE: best linear unbiased estimation
BLUP: best linear unbiased prediction
GBLUP: genomic best linear unbiased prediction
GCA: general combining ability
GEGV: genomically estimated genotypic value
PBLUP: pedigree‐based best linear unbiased prediction
PEGV: phenotype‐based estimate of the genotypic value
S‐All: all hybrid observations of data set
SCA: specific combining abilities
SNP: single nucleotide polymorphism

## 1 INTRODUCTION

The use of genomic selection has led to several modifications in wheat (*Triticum aestivum* L.) breeding programs. One major change is the increase in the value of historical genomic and phenotypic data from previous breeding cycles, which are used in genomic selection to train the genome‐wide prediction models (Storlie & Charmet, 2013). The use of historical genomic data is not difficult as long as the genotyping platform remains largely unchanged or new developments are compatible with those previously used. This was the case, for example, when the 9k single nucleotide polymorphism (SNP) array (Cavanagh et al., 2013) in wheat was updated by the 90k SNP array (Wang et al., 2014). In contrast, using historical phenotypic data to train genome‐wide prediction models can be challenging.

Historical phenotypic data are typically not orthogonal across years (e.g., Storlie & Charmet, 2013) and come from multistage selection programs in which extensive populations are tested in a few environments and a selected fraction are then evaluated in a large number of environments. On the one hand, data from early selection cycles are valuable to train the genome‐wide prediction models because they have a large phenotypic diversity (He et al., 2017); on the other hand, genotypes in early selection cycles are tested in only a few environments, which reduces the heritability and thus the accuracy of the genome‐wide prediction models (He et al., 2017). The optimal design of a wheat breeding program that uses genome‐wide predictions can lead to adjustments to increase the number of environments used for yield testing in early selection cycles as a function of the accuracy of genome‐wide predictions (Longin et al., 2015). Nevertheless, the number of environments is limited by the moderate multiplication coefficient in wheat, limiting the amount of seed available for the next stage. This also hampers the implementation of phenotype imputation, where indirect traits are used to enable sparse yield testing (e.g., Rutkoski et al., 2016; Ward et al., 2019). An important side effect of a small number of environments used in early yield tests is a potential underrepresentation of the diversity of environments, which can lead to a bias in the decomposition of phenotypic variance into effects of genotypes, environments, and their interaction effects (Utz et al., 2000).

Extensive research has been conducted to optimize the design and biometric analyses of plant breeding trials (Cullis et al., 2006; Patterson & Williams, 1976; Smith & Cullis, 2018; Yates, 1940). The use of relationship information has become an important underpinning for crop candidate performance evaluation, as observations of one genotype can inform decisions about related genotypes. Estimations of genotypic values that take known relationships into account can be modeled using best linear unbiased prediction (BLUP). As the genotypic value is therein modeled using one or more random variables, relationship can enter the model in two ways: Either explicit covariance structures for genotype‐related random variables are defined or related genotypes are defined to share common effects. Details about both approaches can be found for example in a review by Piepho et al. (2008). An example of the first approach is the decomposition of the genotypic effect into an additive and dominance component, for which expected covariances result from quantitative genetic theory or are approximated using genomic markers (Alvarez‐Castro & Carlborg, 2006; VanRaden, 2008). The second approach is suitable for evaluation of hybrids, where the genotypic effect of a candidate is decomposed into the sum of the general combining abilities (GCAs) of its two parents and the specific combining ability (SCA) of the specific cross. The GCA of potential parents and thus the most beneficial future crosses are often predicted using crosses to some tester lines, but Seye et al. (2020) recently pointed out that a sparse factorial crossing of parent lines might be superior. This example shows how GCA/SCA‐based models incorporate relationship information without defining a covariance structure of the individual effects. By use of relationship information, BLUP allows an increased flexibility in breeding trial layout as some genotypes can be phenotyped in fewer environments or even not at all (Longin et al., 2015). Computationally, these models are fitted by restricted maximum likelihood estimation (Gilmour et al., 1995) or as a special case of reproducing kernel Hilbert space regression (de los Campos et al., 2009).

**Core Ideas**

- In preliminary trials, evaluating candidates sparsely in more environments improves accuracy.
- A prerequisite for that are biometric models that consider candidate relatedness.
- Joint analysis of concurrent trials can improve accuracy without logistic changes.

The potential to increase the diversity of environments in early yield trials in the context of genomic selection was suggested in a pioneering resampling study using genomic and phenotypic data from a biparental family for barley (*Hordeum vulgare* L.) and maize (*Zea mays* L.; Endelman et al., 2014). The authors observed that genome‐wide prediction models improved when genotypes were distributed across multiple locations rather than testing all entries in one location and concluded that genome‐wide markers in such imbalanced designs establish connectivity by modeling relatedness among entries. Also, Jarquin et al. (2020) demonstrated in two sparse three‐environment maize testcrosses that the prediction accuracy of genome‐wide prediction models can benefit from connecting the environments with common genotypes. However, an in‐depth study on the potential to optimize the design of multienvironmental yield trials in times of genomic selection in diverse populations is lacking. This is very promising, for example, in hybrid breeding with a pronounced degree of relatedness resulting from factorial mating designs.

Our survey is based on a comprehensive published data set of wheat hybrids comprising 1,604 single crosses and their 135 parental lines. We used a resampling strategy to investigate the optimum allocation of resources in multienvironmental field trials assuming restricted budgets. In particular, our objectives were (a) to find the best of several ways to sparsely distribute a fixed number of plots across different environments to increase the accuracy of predicting hybrid performance, (b) to compare several commonly used biometrical models for phenotypic data analysis and identify the one that most accurately estimates the performance of the candidates in such unbalanced trials, and (c) to examine the impact of including a common set of genotypes across all environments.

## 2 MATERIALS AND METHODS

All calculations have been performed using R 4.0.2 (R Core Team, 2020).

### 2.1 Phenotypic data

Our study is based on previously published phenotypic and genomic data of an F_1_ hybrid population resulting from 1,604 out of 1,800 potential single crosses with sufficient seeds for multienvironmental yield trials between 120 female and 15 male winter wheat elite lines that were bred for wheat growing in central Europe (Jiang et al., 2017; Longin et al., 2013; Zhao et al., 2015). Briefly, the hybrids, their parents, and 10 check cultivars were phenotyped for grain yield in 2 year (2012 and 2013) and six locations (Supplemental Figure S4, 11 location‐year combinations in total). The genotypes were divided randomly for each environment into three trials for reasons of field management. The trial designs were partially replicated alpha lattice designs that were connected by 10 replicated check cultivars. The same seeding rate was used for lines and hybrids. Grain yield was adjusted to a moisture concentration of 140 g H_2_O kg^−1^. Further environment details are shown in Supplemental Tables S1 and S2. The parental lines were fingerprinted using a 90K SNP array (Würschum et al., 2013; Zhao, Gowda, et al., 2013). The SNP profiles of the hybrids were derived from the information of the SNP profiles of the parental lines. Our study is based on 16,937 polymorphic SNPs. The phenotypic and genomic data was used in an in silico study to investigate the optimum allocation of resources in multienvironmental field trials assuming restricted budgets. The available hybrid observations of the data set are shown in Supplemental Figure S3.

### 2.2 Phenotypic values adjusted for experimental design effects

As a first step, we analyzed the raw data using the following linear mixed model:

(1) $$\;y_{\mathrm{ijklm}}=g_{i}+e_{j}+t_{\mathrm{jk}}+r_{\mathrm{jkl}}+b_{\mathrm{jklm}}+\varepsilon_{\mathrm{ijklm}}$$

decomposing each observation *y_ijklm_* into a genotypic value *g*_*i*_ for each genotype *i*, an environment effect *e_j_* for each environment (year × location) *j*; a trial effect *t_jk_* for each trial *k*, nested within the environment; a replication effect *r_jkl_* for each replication *l*, nested within the trial; a block effect *b_jklm_* for each block *m*, nested within the replication; and a residual error term for each observation. The genotypic values **g** are modeled as a fixed effect and all other effects as random; that is, **x** ∼ *N*(0; **I**σ*_x_*) for each **x** in **e**, **t**, **r**, **b**, **ε**.

The model was fitted using AsReml (Gilmour et al., 2015). The estimated design effects for **t**, **r**, **b** were then subtracted from the raw data. The resulting data set **y^(d)^**, which has the same size as **y**, was then used for further analysis. The clustering of environments was analyzed in detail in a previous study (See Figure Supplementary Note f‐2 in Zhao et al., 2015) and the absence of distinct clusters was reported.

### 2.3 In silico scenarios of allocation of multienvironmental field trials

As a reference, all hybrid observations of our data set (S‐All) were used to compare different schemes of allocating plots in multienvironmental hybrid grain yield trials. We then defined six in silico scenarios (S1–S6) that contained only 38% of the observations of the S‐All data set (Supplemental Methods). The number of plots corresponds to grain yield assessment of the whole population in about four environments. The yield evaluations in four environments represents a typical scenario for resource allocation for first multienvironmental yield trials in Central European wheat breeding programs.

**FIGURE 1:**
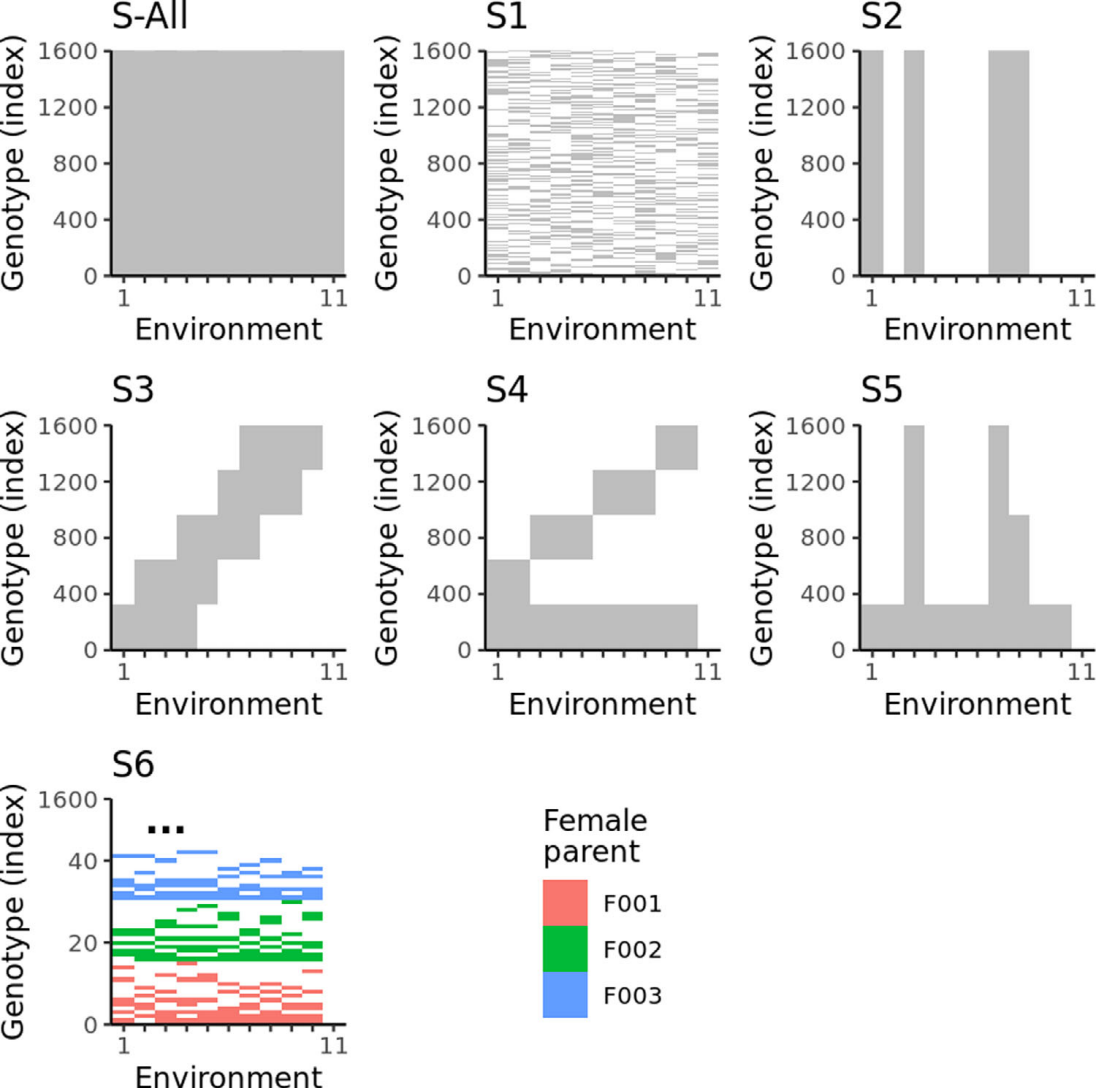
Graphical illustration of how genotypes were assigned to different environments in the six in silico scenarios (S1–S6). A filled square indicates that a genotype was evaluated in an environment. In S6, the female parent of the genotype is indicated by the color. The plot for S6 shows the offspring of the first 3 of the 120 female parents for a clear display of the scenario structure

The first scenarios were designed for conceptual simplicity, modifying as few aspects of the data set as possible at the same time (Figure 1):

- S1: With this scenario we tested the influence of reducing the number of observations in a balanced fashion. It was generated as a random sample from the data set and included data of all 11 environments. The sampling was constrained in that all environments had a similar number of observations, and all genotypes were tested in a similar number of environments.
- S2: With this scenario we tested the influence of a reduced number of environments. It was generated as a balanced data set that consisted of data from four environments randomly selected from the full data set.

The remaining four scenarios were designed to resemble field trials of potential practical relevance to breeders:

- S3: The set of genotypes was divided in five groups of genotypes. Each group was tested in four environments. The groups overlapped with respect to the environments. In total, the scenario included 10 environments.
- S4: One‐fifth of the genotypes were tested in 10 environments. The remaining genotypes were clustered into four groups that were evaluated in separate sets of environments. This pattern may arise from combining trials from early and late stages of two multistage selection programs that are running concurrently, where one program has progressed farther.
- S5: One‐fifth of the genotypes were tested in 10 environments. The remaining genotypes were tested in up to three environments. As for S4, this pattern may arise from combining early‐ and late‐stage trials.
- S6: Half of the genotypes were tested in 4 to 10 environments, the other half in 1 to 3 environments. This pattern was repeated for the hybrid offspring of each female parent so that for every female parent, some offspring were tested in many and some were tested in few environments.

### 2.4 Predicting and estimating reference genotypic values

To have a benchmark for the different scenarios, we obtained estimates or predictions of the performance of the hybrids using the data set S‐All applying two statistical models.

We obtained the best linear unbiased estimations (BLUEs), fitting the following linear model:

(2) $$y_{\mathrm{ijklm}}^{\left(d\right)}=g_{i}+e_{j}+\varepsilon_{\mathrm{ijklm}}$$

where ***y* ^(d)^** is the observations without design effects obtained following Model 1. The estimated/predicted variables are **g**, a vector of fixed genotype effects and **e** and **ε**, vectors of random environment and residual effects, respectively. As in Model 1, the covariance structure of the random effects is the identity matrix.

The model was fitted using AsReml 4 (Gilmour et al., 2015). The BLUEs from the S‐All data set can be interpreted as the purely phenotype‐based estimates of the genotypic value (PEGV).

In a second approach, the following random linear model was fitted that decomposes the effect of the genotype into additive and dominance components. This model is referred to as GBLUP (genomic best linear unbiased prediction) in the following:

(3) $$y_{\mathrm{ijklm}}^{\left(d\right)}=a_{i}+d_{i}+e_{j}+\varepsilon_{\mathrm{ijklm}}$$

The estimated effects include the additive (**a**) and dominance (**d**) genetic effects. The notation of the other effects is analogous to Model 2.

The two genetic effects had covariance structures derived from the SNP profile of the hybrids (**a** ∼ *N*[0, **K_a_** σ*_a_*] and (**d** ∼ *N*[0, **K_d_** σ*_d_*]). The SNP status of the hybrids was calculated as the mean of the respective parents’ SNP status. Then, the covariance structure of the additive effect **K_a_** was calculated according to VanRaden (2008, p. 4116, “first method”). The dominance effect had a covariance structure **K_d_** according to Alvarez‐Castro and Carlborg (2006).

We fitted this model using the R package BGLR (Pérez & de los Campos, 2014) that solves mixed models as a special case of reproducing kernel Hilbert space regression in a Bayesian framework, using a Gibbs sampler. We used 5,000 iterations of which 900 iterations were treated as burn‐in phase. The performance of the Gibbs sampling was checked by calculating the effective sample size of the resulting Markov chains, which corrects the number of iterations with the autocorrelation of the chain, as well as the Geweke (1992) diagnostic, which checks whether the empirical distributions of parameter values at the beginning and the end of the chain are different. The effective sample sizes were high enough to calculate the predicted values by taking the mean of the iteration steps (Supplemental Figure S1A). For the majority of model runs, the Geweke scores were within what was expected from a standard normally distributed random variable. This result conforms to the Markov chains converging to a stationary distribution and thus the burn‐in phase being long enough (Supplemental Figure S1B). We also manually checked the Markov chain trace of the GBLUP of S‐All wherein the convergence to a stationary distribution well within the burn‐in phase can be seen (Supplemental Figure S2).

The obtained predictions, when done on the S‐All data set, can be interpreted as the genomically estimated genotypic value (GEGV).

### 2.5 Statistical models to predict genotypic values from in silico scenarios

We obtained genotypic values from the data sets generated according to the six in silico scenarios described above and S‐All. The statistical models used for this were BLUE (Model 2), GBLUP (Model 3) and two additional models that are described in this section.

In the first model, we obtained the BLUP using the following model with two random terms assuming no specific covariance structure between the genotypes:

(4) $$\;y_{\mathrm{ijklm}}^{\left(d\right)}=g_{i}+e_{j}+\varepsilon_{\mathrm{ijklm}}$$

The model is similar to Model 2 and the same terms are used with the only difference that ***g*** is a random variable with **g** ∼ *N*(0, **I**σ*_a_*).

In the second model, we decomposed the hybrid performance into GCA and SCA effects by fitting the following random effect model:

(5) $$\;y_{\mathrm{ijklm}}^{\left(d\right)}=f_{p\left(i\right)}+m_{q\left(i\right)}+s_{i}+e_{j}+\varepsilon_{\mathrm{ijklm}}$$

As before, the term ***y* ^(d)^** is the observations without design effects (Model 1). All factors are random without covariance structure analogous to Model 1. The parental GCAs are **f** and **m** that have one value for each parent genotype. The functions *p*(*i*) and *q*(*i*) yield the female and male parent index for the hybrid genotype *i*, respectively. The SCA is **s**, with one entry for each hybrid genotype *i*. The remaining effects (environment and residual) are defined similarly to the previous models.

This model takes the pedigree of the hybrids into account implicitly by estimating the influences of their common parents. Thus, we call it pedigree‐based BLUP (PBLUP) in this text. Both Models 4 and 5 do not use SNP data. Nevertheless, Model 5 considers relatedness, as multiple hybrids share the same parent and thus common effects. If Model 5 would be constrained in that σ*_f_* and σ*_m_* must be the same value, the effects **f** and **m** could be coalesced to one random variable whose covariance structure would be the matrix of coefficients of coancestry of the hybrids, assuming a population of unrelated parental genotypes. The Models 4 and 5 were fitted using AsReml 4 (Gilmour et al., 2015)

### 2.6 Evaluating estimates of genotypic values obtained from in silico scenarios

We evaluated the precision of the estimated/predicted genotypic values of the hybrids obtained by Models 2–5 in the Scenarios S1–S6 by correlating these predictions to the reference values, the PEGVs and GEGVs. As each scenario was realized 100 times (Supplemental Methods), for each scenario and model 100 correlation values depending on the data sample result, from which the median and the quartiles were taken.

Moreover, we estimated the reliability with which the best genotypes, according to their PEGV and GEGV, respectively, are found in the best genotypes of the in silico scenarios. We considered for this the 10% best genotypes in terms of PEGV and GEGV, respectively. We then measured the percentage of realizations for each scenario and model in which these genotypes were also among the top 10%. As an example, say 100 hybrid genotypes are studied. From selecting the 10% best by their GEGVs and PEGVs, respectively, two sets of 10 genotypes result. They represent the breeder's choice if the full data set would be available to them. Let us call these two sets G* and P*. To judge a certain scenario and model against these standards, for each of the 100 scenario realizations the best 10 genotypes are taken, resulting in a set of sets S with 100 elements and each element being a set of 10 genotypes. For every genotype in G*, it is now calculated in how many elements of S this genotype is present.

### 2.7 Joint analysis of different series of trials conducted in the same environments

In the Scenarios S4 and S5, not all genotype groups were tested in the same number of environments. Rather, the first four genotype groups were tested in two to three environments while the fifth genotype group was tested in 10 environments.

This situation is similar to an environment in which breeders test two trials where one trial is in a later stage than the other. The late‐stage trial is tested in more environments but comprises fewer genotypes than the earlier trial. We explored whether the accuracy of the early‐stage trial can profit from the inclusion of the concurrent late‐stage trial into a joint analysis.

To answer this question, we split the genotypes of the Scenarios S4 and S5 in two groups, respectively. The genotypes that were measured in two to three environments were designated as early‐stage genotypes. When referring to the early‐stage genotype group of a specific scenario, we use the suffix “e” (e.g., S4e and S5e). Similarly, the genotypes that were measured in 10 environments were designated as late‐stage genotypes, with the suffix “l” (e.g., S4l and S5l).

We focused on the correlations of the yield predictions of the early‐stage genotypes S4e and S5e to the PEGVs and the GEGVs where the predictions were generated with the same collection of models as in the previous section. The data available to the model were either the early‐stage genotypes only, or the early‐stage plus the late‐stage genotypes. Regardless of the data available to the model, only the correlation of the early‐stage genotypes to the PEGVs/GEGVs was considered.

## 3 RESULTS

### 3.1 Definition of genotypic values used as references

**FIGURE 2:**
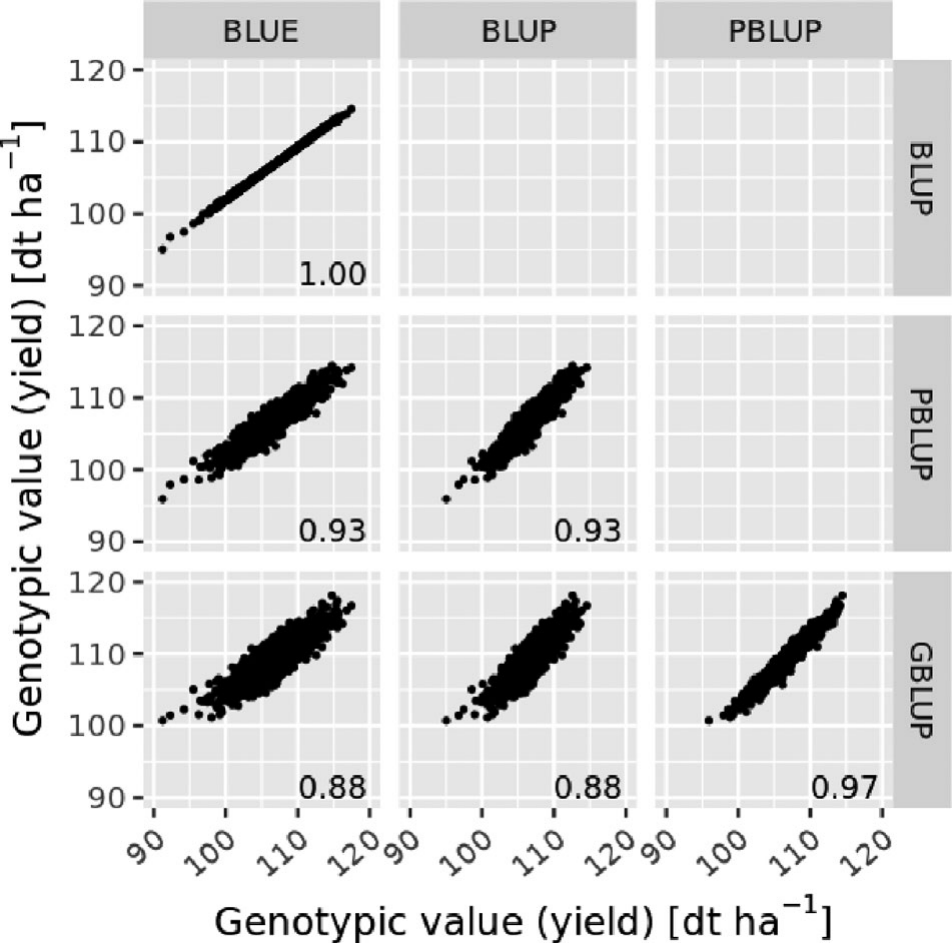
Correlations of hybrid performance predictions/estimates of different models using the S‐All data set. The models are shown on the plot borders. See the model equations in the Materials and Methods section for their definitions: best linear unbiased estimation (BLUE, Model 2), best linear unbiased prediction (BLUP, Model 4), pedigree‐based best linear unbiased estimation (PBLUP, Model 5), genomic best linear unbiased prediction (GBLUP, Model 3). The predictions/estimations of BLUE and GBLUP in this figure are the phenotypically estimated genotypic values (PEGVs) and genomic estimated genotypic values (GEGVs), respectively. The Pearson correlation coefficients are shown in each plot

For the S‐All data set, all biometric models yielded very similar estimations/predictions of the hybrid performances (Figure 2). The PEGVs and GEGVs, estimations/predictions resulting from the BLUE and GBLUP models, respectively, correlated to each other with 0.88. The BLUPs correlated very strongly to the PEGVs (>0.99). Moreover, PBLUP showed correlations of 0.93 to 0.97 to the predictions from the other models. Summarizing, the values from BLUE and GBLUP form the two extremes of the studied predictions as their correlations to each other was the lowest of all models. They therefore are complementary approaches to define the genotypic values used as references for the in silico scenario predictions. This is elaborated in more detail in the discussion.

Genotypic values obtained with the PBLUP and GBLUP models were more strongly associated with the reference values than those of the BLUE and BLUP models

Unless otherwise stated, we focused on the median of correlations between estimates/predictions and genotypic reference values for comparisons below. Also, we refer to the genotypic value predictions/estimations of a model from an in silico scenario by writing the model name with an “s” appended (e.g., “BLUEs”).

Across all scenarios, the BLUEs never outperformed the BLUPs. Depending on the choice of reference values and scenario, the correlations for Scenarios S4 to S6 were 0.03 to 0.06 lower for the BLUEs than for the BLUPs and approximately the same for Scenarios S1 to S3.

**FIGURE 3:**
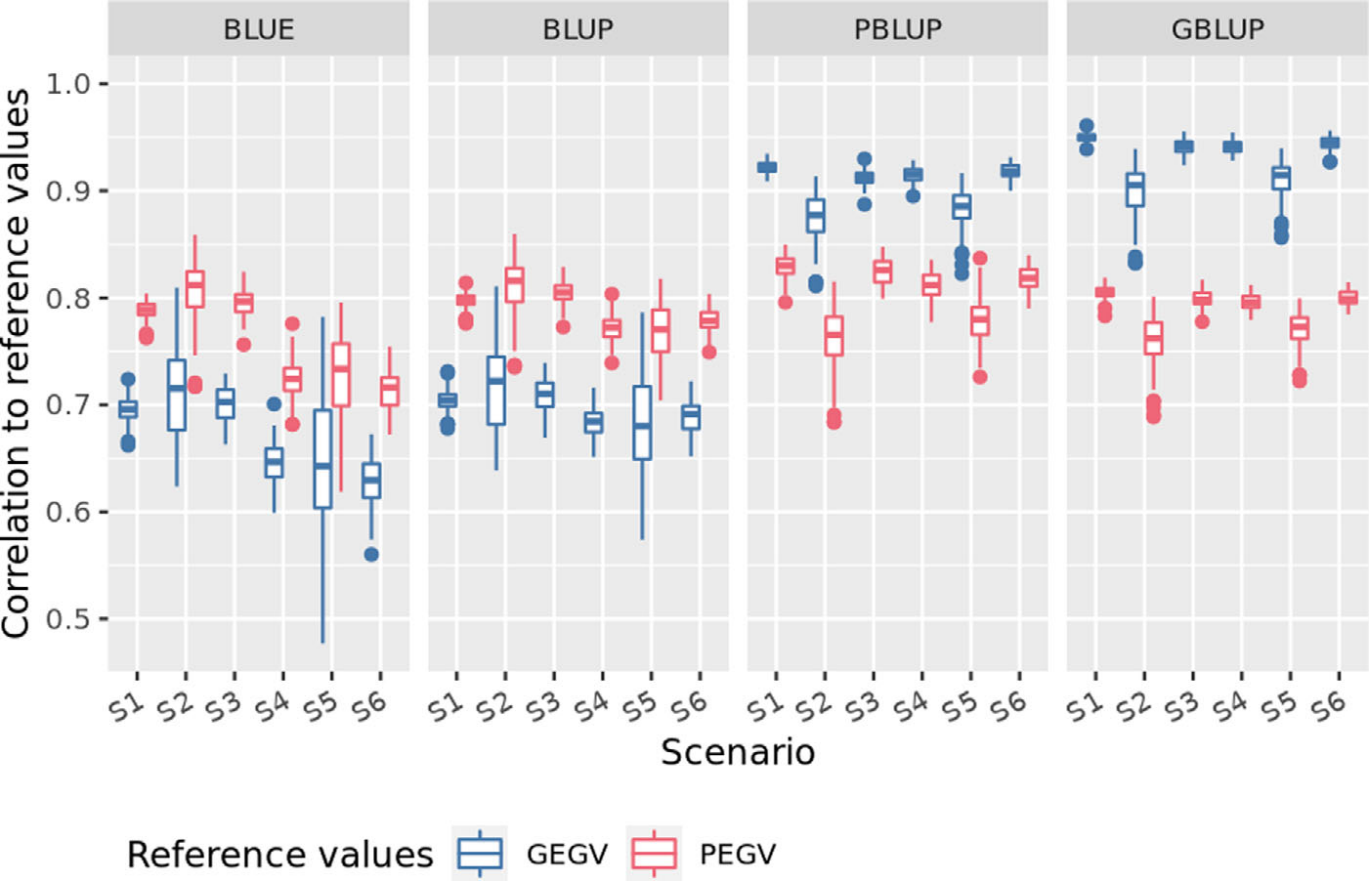
Correlations of the hybrid performance predictions/estimations of different models and scenarios to the phenotypically estimated genotypic values (PEGVs) and genomic estimated genotypic values (GEGVs) (colors). The models are shown above the plots. For the structure of the scenarios, see Figure 1. Each plot shows the correlations resulting from one model in the different scenarios. Each box plot summarizes 100 realizations. BLUE, best linear unbiased estimation; BLUP, best linear unbiased prediction; PBLUP, pedigree‐based best linear unbiased estimation; GBLUP, genomic best linear unbiased prediction

Within the scenarios, the predictions of the different biometric models correlated more strongly with the reference values of the model to which they are conceptually most similar: The BLUEs and BLUPs from the scenarios are stronger correlated to the PEGVs (0.72–0.82) than to the GEGVs (0.63–0.70). Conversely, the PBLUPs and GBLUPs are more similar to the GEGVs (0.88–0.95) than to the PEGVs (0.76–0.83; Figure 3 and Table 1).

**TABLE 1:** Median correlations of the hybrid performance predictions/estimations of the scenarios and models to the reference values (“Ref.”)

**Ref**.	**Scenario**	**Model order**	**BLUE**	**BLUP**	**PBLUP**	**GBLUP**
GEGV	S1	BLUE = BLUP < PBLUP < GBLUP	0.70	0.70	0.92	0.95
GEGV	S2	BLUE = BLUP < PBLUP < GBLUP	0.72	0.72	0.88	0.91
GEGV	S3	BLUE = BLUP < PBLUP < GBLUP	0.70	0.71	0.91	0.94
GEGV	S4	BLUE < BLUP < PBLUP < GBLUP	0.65	0.68	0.92	0.94
GEGV	S5	BLUE < BLUP < PBLUP < GBLUP	0.64	0.68	0.89	0.91
GEGV	S6	BLUE < BLUP < PBLUP < GBLUP	0.63	0.69	0.92	0.95
PEGV	S1	BLUE = BLUP = GBLUP < PBLUP	0.79	0.80	0.83	0.81
PEGV	S2	GBLUP = PBLUP < BLUE = BLUP	0.81	0.82	0.77	0.76
PEGV	S3	BLUE = GBLUP = BLUP < PBLUP	0.80	0.81	0.83	0.80
PEGV	S4	BLUE < BLUP < GBLUP < PBLUP	0.72	0.77	0.81	0.80
PEGV	S5	BLUE < BLUP = GBLUP = PBLUP	0.73	0.77	0.78	0.77
PEGV	S6	BLUE < BLUP < GBLUP < PBLUP	0.72	0.78	0.82	0.80

*Note*. The column „Model order“ summarizes which models lead to higher correlations for the same scenario. Any difference above 0.01 is denoted by “<”, smaller differences by “=”. BLUE, best linear unbiased estimation; BLUP, best linear unbiased prediction; PBLUP, pedigree‐based best linear unbiased estimation; GBLUP, genomic best linear unbiased prediction; GEGV, genomic estimated genotypic value; PEGV, phenotypically estimated genotypic value.

Regardless, averaged across all scenarios, PBLUPs or GBLUPs showed a higher correlation to the reference values PEGV and GEGV than BLUEs or BLUPs: the correlation to the GEGVs is about 0.2 higher for the PBLUPs and GBLUPs than for the BLUPs and BLUEs. The correlations to the PEGVs, on the other hand, differed less as a function of the model, but again, in most cases PBLUPs and GBLUPs correlated equally well or stronger to the PEGV in most of the cases than predictions by BLUE or BLUP (difference 0.00–0.08) The only case where this model ranking was less clear was in Scenario S2, in which BLUEs and BLUPs predictions correlated with the PEGVs with 0.81 on average, whereas the PBLUPs and GBLUPs predictions correlated with 0.76 on average. Considering the GEGV correlation, however, the GBLUPs or PBLUPs of Scenario S2 performed about 0.2 higher off than the other two models. In conclusion, in our study, the genomic models PBLUP and GBLUP were at an advantage against BLUE and BLUP.

### 3.2 Balanced environmental sampling boosts the accuracy of PBLUP and GBLUP

The choice of the genotypic reference value did not affect which in silico scenario yielded the best correlations to the reference values for a certain model (Figure 3). In contrast, the choice of biometric model did impact the optimal choice of scenarios. Here, the results of BLUE and BLUP were similar, and the results of PBLUP and GBLUP were also similar.

The in silico Scenario S2 was, on average, the scenario with the highest correlations of the BLUEs and BLUPs to the genotypic reference values. However, there was a high variation among the individual realizations. The predictions for the Scenarios S1 and S3 had only slightly lower correlations (differences of the medians of 0.02 or less) but the variance among realizations was much lower for these scenarios. The BLUPs and BLUEs from the remaining Scenarios S4 to S6 were 0.01 to 0.10 lower than those from the other scenarios (Figure 3).

For the PBLUPs and GBLUPs, the Scenario S1 showed the highest correlation for both reference values than the predictions of the other scenarios (Figure 3). However, Scenarios S3, S4, and S6 also nearly reached this level with differences between the correlations of less than 0.02. The PBLUPs and GBLUPs of the Scenarios S2 and S5 were less correlated with the reference than the predictions from S1, S3, S4, and S6 with differences ranging from 0.02 to 0.06.

In summary, the performance of the BLUE and BLUP benefited when the genotypes were tested in a comparable number of environments (Scenarios S1, S2, and S3). In contrast, the PBLUP and GBLUP benefited when each environment had a comparable number of observations, adequately characterizing each environment (Scenarios S1, S3, S4, and S6).

### 3.3 Selection by PBLUPs or GBLUPs had the highest average probability to recover the top genotypes

**FIGURE 4:**
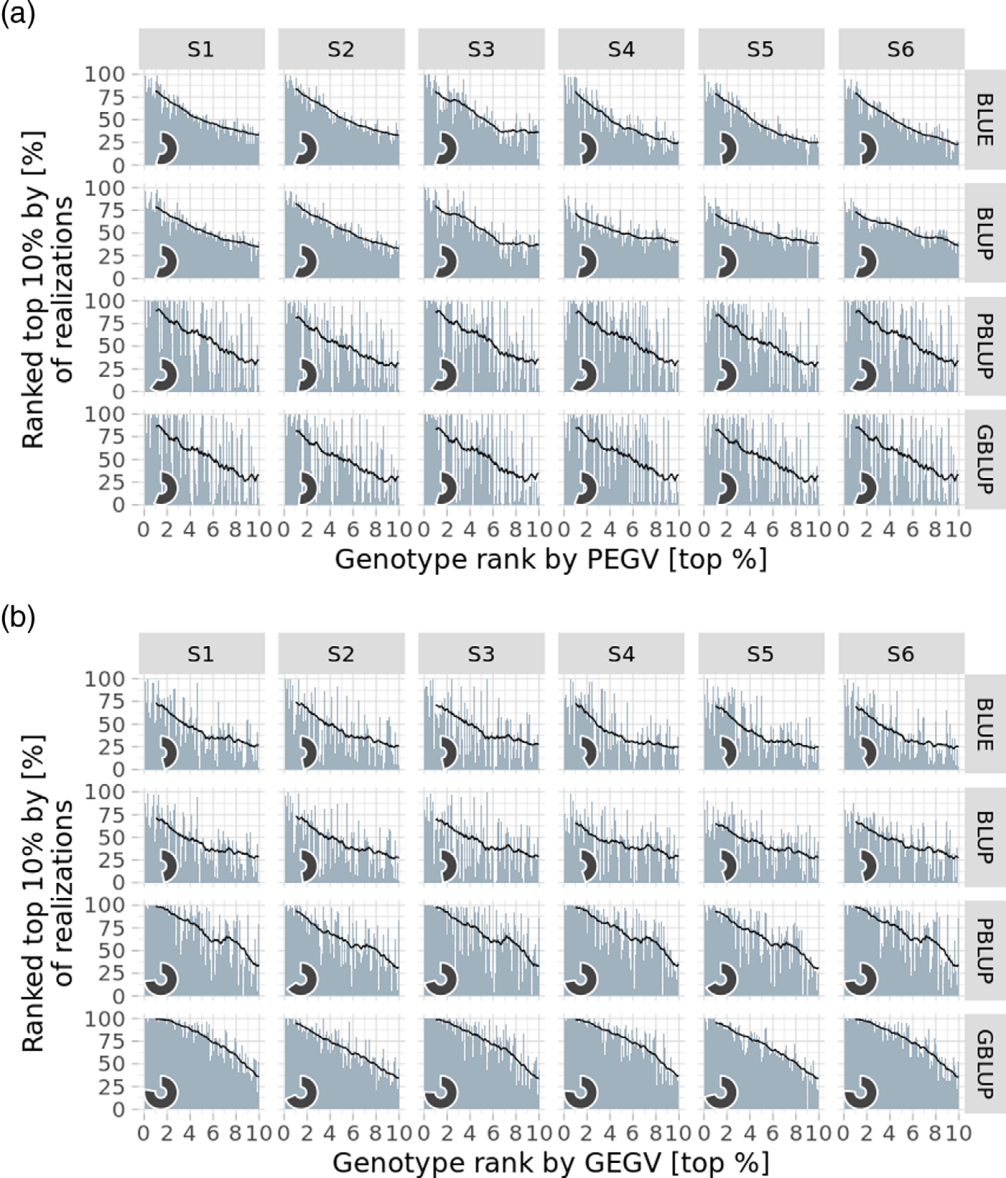
Fractions of realizations in which the top 10% genotypes by yield phenotypically estimated genotypic values (PEGV) (a) or genomic estimated genotypic value (GEGV) (b) are found among the top 10% of the predictions from different scenarios and models (fraction of realizations) as well. Each bar corresponds to one genotype, the circular bar in the plot corner shows the average amount of top 10% genotypes that were recovered to be in the top 10% of scenario predictions. See Table 2 for numeric values. The black line shows the average within a moving window of 2% (32 genotypes). BLUE, best linear unbiased estimation; BLUP, best linear unbiased prediction; PBLUP, pedigree‐based best linear unbiased estimation; GBLUP, genomic best linear unbiased prediction

Up to this point, we have evaluated the merits of the models and scenarios by looking at the entire population. However, breeders are more interested in finding the top or a proportion of the best genotypes. For example, if we consider the best 10% of genotypes, the following picture emerges. (a) Taking PEGV as reference genotypic values, no large differences were found between the biometric models for finding the best 10% genotypes. The values varied between 49 and 60% (Figure 4A and Table 2). Except for Scenario S2, PBLUP had slight advantages of 3–5 percentage points compared with BLUP. When the selection intensity was increased by, for example, to the top 2%, 82–90% were identified on average across the scenarios by PBLUP or GBLUP, but only 71–84% by BLUP (see the moving window averages in Figure 4A). (b) When considering the GEGV as reference values, the picture was different, and the choice of the biometric model strongly influenced the results. The recovery rate of the best 10% of genotypes was 45–48% when analyzed with BLUP, whereas the recovery rate was markedly higher with 65–78% for the PBLUP and GBLUP models (Figure 4B and Table 2). Summarizing, the PBLUP and GBLUP models were superior to BLUP and BLUE in that they either yielded a higher rate of recovered top 10% genotypes from the scenarios or, in cases where this figure was similar between the models, the advantage of the PBLUP and GBLUP models over BLUP increased when the group of top genotypes was defined increasingly smaller.

**TABLE 2:** Mean percentage of realizations in which the top 10% of genotypes by PEGV or GEGV, respectively, were ranked among the top 10% of scenario (S) predictions

**Reference**	**Model**	**S1**	**S2**	**S3**	**S4**	**S5**	**S6**
PEGV	BLUE	54.8	56.5	55.5	49.0	48.8	49.2
PEGV	BLUP	55.5	56.9	56.1	53.4	53.1	55.7
PEGV	PBLUP	60.4	52.9	59.4	59.4	55.6	59.8
PEGV	GBLUP	56.4	51.9	54.7	55.9	53.2	56.2
GEGV	BLUE	46.3	47.1	46.9	42.0	42.4	42.5
GEGV	BLUP	46.8	48.0	47.7	45.2	45.0	46.4
GEGV	PBLUP	73.0	65.4	71.2	72.2	66.8	72.5
GEGV	GBLUP	78.0	68.5	75.6	76.6	70.5	76.6
Mean		58.9	55.9	58.4	56.7	54.4	57.4

*Note*. BLUE, best linear unbiased estimation; BLUP, best linear unbiased prediction; PBLUP, pedigree‐based best linear unbiased estimation; GBLUP, genomic best linear unbiased prediction; GEGV, genomic estimated genotypic value; PEGV, phenotypically estimated genotypic value.

Comparing different in silico scenarios, which reflect different allocations of plots in a multienvironmental field trial, the predictions from Scenarios S1 and S3 were the most consistent, as they showed high proportions of recovered top genotypes compared with the predictions of other scenarios (Table 2). In Scenario S2, the aforementioned advantage of GBLUP and PBLUP over BLUP and BLUE was the smallest. In recovering the best PEGV genotype, BLUP outperformed PBLUP by 4 percentage points in S2.

However, in summary, the choice of the scenario was less important than the choice of the biometric model. The PBLUP and GBLUP models were superior to the BLUE and BLUP in that they either yielded a higher rate of recovered top 10% genotypes from the scenarios or, in cases where this figure was similar between the models, the advantage of the PBLUP and GBLUP models over BLUP increased when the top genotype group was defined increasingly smaller.

### 3.4 An early‐stage trial can profit from joint analysis with related, later‐stage trials

**FIGURE 5:**
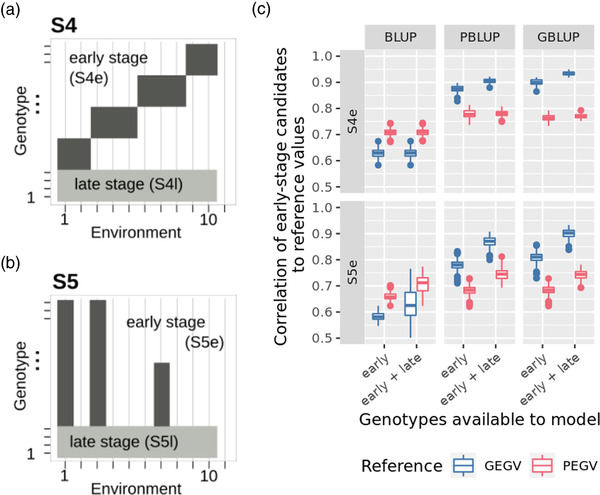
(a,b): Schematics of the early‐ and late‐stage genotypes in Scenarios S4 and S5. (c): Correlations of the hybrid predictions from the early‐stage genotypes of Scenarios S4 and S5 (thus suffixed with “e”) to the PEGVs or GEGVs. The two plot rows show the correlations for the two genotype groups S4e and S5e (see Methods section) and the three plot columns show the results for each of the models used to estimate candidate performance. The horizontal plot axis lists the different sets of genotypes available to the model, detailed in (a) and (b)

The correlation of the predictions of the early‐stage genotypes S4e and S5e to the two reference values was higher when the early‐ and late‐stage genotypes were jointly analyzed than when only the early‐stage genotypes were used. This joint analysis of early‐ and late‐stage genotypes lead to higher correlations to the reference values for S5e candidates (0.04–0.09 higher) than for the S4e candidates (0.00–0.04 higher, Figure 5C). Dissecting this trend further, the correlations to the GEGVs increased for all cases except the S4e BLUPs, while the correlation to the PEGVs increased only for S5e whereas PEGV correlations of S4e candidates remained unchanged. However, jointly using early‐ and late‐stage genotypes instead of early‐stage genotypes alone never led to inferior results, so averaging across the two references, using early‐ and late‐stage genotypes jointly, was the safer choice to achieve high correlations of early‐stage genotypes to the reference values.

## 4 DISCUSSION

### 4.1 The choice of proper reference values for the genotypic values

We used two different reference values, PEGVs and GEGVs, to compare different arrangements of field trials analyzed with four popular biometric models. This raises the question of which of the two reference values approximates the genotypic value more precisely and is therefore more informative for a breeder. The model underlying PEGVs is also used by the Federal Variety Office in Germany for candidate evaluations, and therefore a formal argument is that the PEGVs are of direct interest to breeders. The PEGVs reflect estimates of the genotypic values based on only a few assumptions, such as, for example, randomly distributed residual errors, but disregard information on genetic relationships among candidates and implies that all genotypes have a homogeneous genotypic variance and are independent. The latter is particularly important for hybrid wheat because factorial mating designs (Bernardo, 2010, p. 123), as used, for example, in our study to generate the hybrids, result in extensive half‐sib families.

In contrast to the PEGVs, the predictions of GEGVs consider the relatedness between genotypes. Conceptually, the hybrid performance is decomposed in the applied model into a component of the breeding value and the dominance deviation, assuming that epistatic and genotype × environment effects do not play a role (Falconer, 1989, p. 125). The GBLUP model is equivalent to ridge regression BLUP for large numbers of SNPs (Habier et al., 2007), which means that GEGVs assume equal contributions of all loci to the genetic variance (Meuwissen et al., 2001) and effect size estimates are shrinked stronger for markers with low minor allele frequency (Gianola, 2013). A further assumption made in the decomposition of the hybrid performance is that the two genetic effects are orthogonally defined, which unfortunately is not always the case due to linkage disequilibrium (Alvarez‐Castro & Carlborg, 2006). Furthermore, the prediction of the hybrid performance uses variance components of the genetic effects (Bernardo, 2010, p. 283). Estimations of variance components, especially in small data sets, can be quite erroneous (Huang & Mackay, 2016) and, thus, influence the prediction of the hybrid performance. In summary, GEGVs use more information compared with PEGVs, but they are also based on numerous assumptions. Therefore, the superiority of GEGVs over PEGVs depends very much on whether the information used is accurate and whether the assumptions can be made. Thus, the question of superiority cannot be definitely answered and, as a result, the comparison between different arrangements of field trials analyzed with four different biometric models was performed on both GEGV and PEGV.

### 4.2 PBLUP and GBLUP outperform BLUE and BLUP under most circumstances

Considering the ambiguity described above regarding the optimal reference values, the PBLUP and GBLUP models stand out as analysis models that provide stable and superior results in terms of correlations in all tested scenarios except S2 (Figure 3, Figure 4). For all other scenarios, the advantage of PBLUP and GBLUP over BLUP and BLUE in predicting the true genotypic values depends on where the true values fall in the spectrum between PEGVs and GEGVs. Assuming that the true values would be best captured by the PEGVs, the only settings that outperform the “classical” approach, S2 analyzed with BLUP, are S1 and S3, analyzed by PBLUP. The better the GEGVs capture the true genotypic values, the larger the number of scenarios grows in which PBLUP and GBLUP estimate the true values better than BLUP and BLUE.

Further refining the winner between PBLUP and GBLUP is not possible in this study. The PBLUP prediction accuracies are influenced less by the reference value choice so that, within a scenario, PBLUP outperforms GBLUP in terms of PEGV correlation but is inferior in terms of GEGV correlation. This pattern is essentially reflected when assessing the fraction of top genotypes that are also recovered as such by the scenario predictions (Figure 4). When comparing predictions from these two models with respect to their GEGV correlation one must consider that GBLUP is also the underlying model of the GEGVs. However, the extent to which this influences the result cannot be answered in this study.

### 4.3 Scenario S3 represents an interesting alternative to balanced testing of genotypes in a subset of environments

Scenario S2 represents a current standard of a preliminary yield trial for many breeding programs (Zhao, Zeng, et al., 2013). It is interesting to note that for both reference values, one of the alternative scenarios (S1, S3, or S6) in which the genotypes are distributed over more environments leads to an improved prediction accuracy and, moreover, a significantly reduced variability in the estimations or predictions of the genotypic values (Figure 3). In this context, the advantage of the above alternative scenarios over a classical balanced experiment in a few environments (S2) becomes much higher if one uses the GEGVs instead of the PEGVs as reference values. Therefore, our results indicate that wheat breeders could improve their preliminary yield trials by changing the design of preliminary yield trials. As an alternative design, Scenario S3 seems to us to be particularly suitable, because it provided both high accuracies in the estimations/predictions and yet showed low variability resulting on the used data sample. Also, as the genotypes are not completely randomly distributed like in the case of Scenario S1, it might be easier to implement from a logistic standpoint. Finally, if implementation of S3 is not possible, and the preliminary trials have to be tested in the same few environments, some of the benefits of pedigree‐based or genomic modeling could still be harvested by including candidates that are related but tested in different trials and environments in a joint phenotypic analysis (Figure 5).

### 4.4 Conclusions and Outlook

The final decision on the design of multienvironment hybrid wheat yield trials also depends on cost scenarios where increasing the number of locations could cause additional costs besides the cost per plot. However, a breeder might have infrastructure already present in many environments when advanced trials are evaluated there. Therefore, implementing sparse preliminary testing might not mean using additional environments but redistributing plots of preliminary trials to environments that are already in use, albeit only for advanced and not preliminary trials. Our results clearly demonstrated the utility for optimizing the design of multienvironment hybrid wheat yield trials in times of genomic selection. However, our results are not limited to hybrid breeding programs but are also of interest for line breeding. The study of Endelman et al. (2014) already pioneered extended environmental sampling in a design similar to our Scenario S1. They tested candidates from extensive biparental populations of maize and barley in a sparse fashion in up to three environments. In this case, genome‐wide markers established the connectivity between full sibs and allowed better control for interaction effects between genotypes and environment. In principle, this is also expected for comprehensive diversity panels of wheat inbred line breeding programs, as high prediction accuracies have also been reported in such situations (He et al., 2016).

## AUTHOR CONTRIBUTIONS

Moritz Lell: Conceptualization; Formal analysis; Investigation; Methodology; Visualization; Writing‐original draft. Jochen Reif: Conceptualization; Funding acquisition; Methodology; Project administration; Supervision; Writing‐review & editing. Yusheng Zhao: Conceptualization; Methodology; Supervision; Writing‐review & editing.

## CONFLICT OF INTEREST

The authors declare no conflict of interest.

## Supporting information

Supplemental Figure S1: Statistics of the Gibbs sampler chains from the GBLUP models to judge sampler convergence.
Supplemental Figure S2: Trace plot of the GBLUP model fit of the ‘S‐All’ dataset (see methods part), estimated using Gibbs sampling.
Supplemental Figure S3: Observations of hybrid genotypes in the hybrid wheat data set used in this study.
Supplemental Figure S4: Geographical locations of tested locations in Germany (latitude, longitude): Adenstedt (52.3° N, 10.1° E), Böhnshausen (51.9° N, 11.0° E), Hadmersleben (52.0° N, 11.3° E), Harzhof (54.4° N, 9.9° E), Hohenheim (48.7° N, 9.2° E), Seligenstadt (49.9° N, 10.1° E).
Supplemental Figure S5: Overview characteristics of environments.
Supplemental Table S1: Minimum, maximum and mean yield observed in each of the study environments.
Supplemental Table S2: Variance components, repeatabilities of the study environments (location times year combinations), as well as heritability.

## References

[tpg220150-bib-0001] Alvarez‐Castro, J. M. , & Carlborg, O. (2006). A unified model for functional and statistical epistasis and its application in quantitative trait loci analysis. Genetics, 176(2), 1151—1167. 10.1534/genetics.106.067348 PMC1894581 17409082

[tpg220150-bib-0002] Bernardo, R. (2010). Breeding for quantitative traits (2nd ed.). Stemma Press.

[tpg220150-bib-0003] de los Campos, G. , Naya, H. , Gianola, D. , Crossa, J. , Legarra, A. , Manfredi, E. , Weigel, K. , & Cotes, J. M. (2009). Predicting quantitative traits with regression models for dense molecular markers and pedigree. Genetics, 182(1), 375–385. 10.1534/genetics.109.101501 19293140 PMC2674834

[tpg220150-bib-0004] Cavanagh, C. R. , Chao, S. , Wang, S. , Huang, B. E. , Stephen, S. , Kiani, S. , Forrest, K. , Saintenac, C. , Brown‐Guedira, G. L. , Akhunova, A. , See, D. , Bai, G. , Pumphrey, M. , Tomar, L. , Wong, D. , Kong, S. , Reynolds, M. , Lopez da Silva, M. , Bockelman, H. , Talbert, L. , … Akhunov, E. (2013). Genome‐wide comparative diversity uncovers multiple targets of selection for improvement in hexaploid wheat landraces and cultivars. Proceedings of the National Academy of Sciences of the United States of America, 110(20), 8057–8062. 10.1073/pnas.1217133110 23630259 PMC3657823

[tpg220150-bib-0005] Cullis, B. R. , Smith, A. B. , & Coombes, N. E. (2006). On the design of early generation variety trials with correlated data. Journal of Agricultural, Biological, and Environmental Statistics, 11(4), 381–393. 10.1198/108571106x154443

[tpg220150-bib-0006] Endelman, J. B. , Atlin, G. N. , Beyene, Y. , Semagn, K. , Zhang, X. , Sorrells, M. E. , & Jannink, J.‐L. (2014). Optimal design of preliminary yield trials with genome‐wide markers. Crop Science, 54(1), 48–59. 10.2135/cropsci2013.03.0154

[tpg220150-bib-0007] Falconer, D. S. (1989). Introduction to quantitative genetics. Longman.

[tpg220150-bib-0008] Geweke, J. (1992). Evaluating the accuracy of sampling‐based approaches to the calculation of posterior moments. In J. M. Bernardo , J. O. Berger , A. P. Dawid , & A. F. M. Smith (Eds.), Bayesian statistics (Vol. 4, pp. 625–631). Clarendon Press.

[tpg220150-bib-0009] Gianola, D. (2013). Priors in whole‐genome regression: The Bayesian alphabet returns. Genetics, 194(3), 573–596. 10.1534/genetics.113.151753 23636739 PMC3697965

[tpg220150-bib-0010] Gilmour, A. R. , Gogel, B. J. , Cullis, B. R. , Welham, S. J. , & Thompson, R. (2015). ASReml user guide release 4.1 functional specification. VSN International Ltd. https://www.vsni.co.uk

[tpg220150-bib-0011] Gilmour, A. R. , Thompson, R. , & Cullis, B. R. (1995). Average information REML: An efficient algorithm for variance parameter estimation in linear mixed models. Biometrics, 51(4). 1440–1450 10.2307/2533274

[tpg220150-bib-0012] Habier, D. , Fernando, R. L. , & Dekkers, J. C. M. (2007). The impact of genetic relationship information on genome‐assisted breeding values. Genetics, 177(4), 2389–2397. 10.1534/genetics.107.081190 18073436 PMC2219482

[tpg220150-bib-0013] He, S. , Reif, J. C. , Korzun, V. , Bothe, R. , Ebmeyer, E. , & Jiang, Y. (2017). Genome‐wide mapping and prediction suggests presence of local epistasis in a vast elite winter wheat populations adapted to Central Europe. Theoretical and Applied Genetics, 130(4), 635–647. 10.1007/s00122-016-2840-x 27995275

[tpg220150-bib-0014] He, S. , Schulthess, A. W. , Mirdita, V. , Zhao, Y. , Korzun, V. , Bothe, R. , Ebmeyer, E. , Reif, J. C. , & Jiang, Y. (2016). Genomic selection in a commercial winter wheat population. Theoretical and Applied Genetics, 129(3), 641–651. 10.1007/s00122-015-2655-1 26747048

[tpg220150-bib-0015] Huang, W. , & Mackay, T. F. C. (2016). The genetic architecture of quantitative traits cannot be inferred from variance component analysis. PLOS Genetics, 12(11), e1006421. 10.1371/journal.pgen.1006421 27812106 PMC5094750

[tpg220150-bib-0016] Jarquin, D. , Howard, R. , Crossa, J. , Beyene, Y. , Gowda, M. , Martini, J. W. R. , Pazaran, G. C. , Burgueño, J. , Pacheco, A. , Grondona, M. , Wimmer, V. , & Prasanna, B. M. (2020). Genomic prediction enhanced sparse testing for multi‐environment trials. G3 Genes|Genomes|Genetics, 10(8), 2725–2739. 10.1534/g3.120.401349 32527748 PMC7407457

[tpg220150-bib-0017] Jiang, Y. , Schmidt, R. H. , Zhao, Y. , & Reif, J. C. (2017). A quantitative genetic framework highlights the role of epistatic effects for grain‐yield heterosis in bread wheat. Nature Genetics, 49(12), 1741–1746. 10.1038/ng.3974 29038596

[tpg220150-bib-0018] Longin, C. F. H. , Gowda, M. , Mühleisen, J. , Ebmeyer, E. , Kazman, E. , Schachschneider, R. , Schacht, J. , Kirchhoff, M. , Zhao, Y. , & Reif, J. C. (2013). Hybrid wheat: Quantitative genetic parameters and consequences for the design of breeding programs. Theoretical and Applied Genetics, 126(11), 2791–2801. 10.1007/s00122-013-2172-z 23913277

[tpg220150-bib-0019] Longin, C. F. H. , Mi, X. , & Würschum, T. (2015). Genomic selection in wheat: Optimum allocation of test resources and comparison of breeding strategies for line and hybrid breeding. Theoretical and Applied Genetics, 128(7), 1297–1306. 10.1007/s00122-015-2505-1 25877519

[tpg220150-bib-0020] Meuwissen, T. H. , Hayes, B. J. , & Goddard, M. E. (2001). Prediction of total genetic value using genome‐wide dense marker maps. Genetics, 157(4), 1819. 10.1093/genetics/157.4.1819 11290733 PMC1461589

[tpg220150-bib-0021] Patterson, H. D. , & Williams, E. R. (1976). A new class of resolvable incomplete block designs. Biometrika, 63(1), 83–92. 10.1093/biomet/63.1.83

[tpg220150-bib-0022] Pérez, P. , & de los Campos, G. (2014). Genome‐wide regression and prediction with the BGLR statistical package. Genetics, 198(2), 483–495. 10.1534/genetics.114.164442 25009151 PMC4196607

[tpg220150-bib-0023] Piepho, H. P. , Möhring, J. , Melchinger, A. E. , & Büchse, A. (2008). BLUP for phenotypic selection in plant breeding and variety testing. Euphytica, 161(1‐2), 209–228. 10.1007/s10681-007-9449-8

[tpg220150-bib-0024] Rutkoski, J. , Poland, J. , Mondal, S. , Autrique, E. , Pérez, L. G. , Crossa, J. , Reynolds, M. , & Singh, R. (2016). Canopy temperature and vegetation indices from high‐throughput phenotyping improve accuracy of pedigree and genomic selection for grain yield in wheat. G3 Genes|Genomes|Genetics, 6(9), 2799–2808. 10.1534/g3.116.032888 27402362 PMC5015937

[tpg220150-bib-0025] Seye, A. I. , Bauland, C. , Charcosset, A. , & Moreau, L. (2020). Revisiting hybrid breeding designs using genomic predictions: Simulations highlight the superiority of incomplete factorials between segregating families over topcross designs. Theoretical and Applied Genetics, 133(6), 1995–2010. 10.1007/s00122-020-03573-5 32185420

[tpg220150-bib-0026] Smith, A. B. , & Cullis, B. R. (2018). Plant breeding selection tools built on factor analytic mixed models for multi‐environment trial data. Euphytica, 214(8), 143. 10.1007/s10681-018-2220-5

[tpg220150-bib-0027] Storlie, E. , & Charmet, G. (2013). Genomic selection accuracy using historical data generated in a wheat breeding program. The Plant Genome, 6(1). 10.3835/plantgenome2013.01.0001

[tpg220150-bib-0028] R Core Team (2020). R: A language and environment for statistical computing. R Foundation for Statistical Computing. https://www.r‐project.org

[tpg220150-bib-0029] Utz, H. F. , Melchinger, A. E. , & Schön, C. C. (2000). Bias and sampling error of the estimated proportion of genotypic variance explained by quantitative trait loci determined from experimental data in maize using cross validation and validation with independent samples. Genetics, 154(4), 1839–1849. 10.1093/genetics/154.4.1839 10866652 PMC1461020

[tpg220150-bib-0030] VanRaden, P. M. (2008). Efficient methods to compute genomic predictions. Journal of Dairy Science, 91(11), 4414–4423. 10.3168/jds.2007-0980 18946147

[tpg220150-bib-0031] Wang, S. , Wong, D. , Forrest, K. , Allen, A. , Chao, S. , Huang, B. E. , Maccaferri, M. , Salvi, S. , Milner, S. G. , Cattivelli, L. , Mastrangelo, A. M. , Whan, A. , Stephen, S. , Barker, G. , Wieseke, R. , Plieske, J. , International Wheat Genome Sequencing Consortium , Lillemo, M. , Mather, D. , Appels, R. , … Akhunov, E. (2014). Characterization of polyploid wheat genomic diversity using a high‐density 90∼000 single nucleotide polymorphism array. Plant Biotechnology Journal, 12(6), 787–796. 10.1111/pbi.12183 24646323 PMC4265271

[tpg220150-bib-0032] Ward, B. P. , Brown‐Guedira, G. , Tyagi, P. , Kolb, F. L. , Sanford, D. A. , Sneller, C. H. , & Griffey, C. A. (2019). Multienvironment and multitrait genomic selection models in unbalanced early‐generation wheat yield trials. Crop Science, 59(2), 491–507. 10.2135/cropsci2018.03.0189

[tpg220150-bib-0033] Würschum, T. , Langer, S. M. , Longin, C. F. H. , Korzun, V. , Akhunov, E. , Ebmeyer, E. , Schachschneider, R. , Schacht, J. , Kazman, E. , & Reif, J. C. (2013). Population structure, genetic diversity and linkage disequilibrium in elite winter wheat assessed with SNP and SSR markers. Theoretical and Applied Genetics, 126(6), 1477–1486. 10.1007/s00122-013-2065-1 23429904

[tpg220150-bib-0034] Yates, F. (1940). The recovery of inter‐block information in balanced incomplete block designs. Annals of Eugenics, 10(1), 317–325. 10.1111/j.1469-1809.1940.tb02257.x

[tpg220150-bib-0035] Zhao, Y. , Gowda, M. , Würschum, T. , Friedrich, C. , Longin, H. , Korzun, V. , Kollers, S. , Schachschneider, R. , Zeng, J. , Fernando, R. , Dubcovsky, J. , & Reif, J. C. (2013). Dissecting the genetic architecture of frost tolerance in central European winter wheat. Journal of Experimental Botany, 64(14), 4453–4460. 10.1093/jxb/ert259 24006418 PMC3808325

[tpg220150-bib-0036] Zhao, Y. , Li, Z. , Liu, G. , Jiang, Y. , Maurer, H. P. , Würschum, T. , Mock, H.‐P. , Matros, A. , Ebmeyer, E. , Schachschneider, R. , Kazman, E. , Schacht, J. , Gowda, M. , Longin, C. F. H. , & Reif, J. C. (2015). Genome‐based establishment of a high‐yielding heterotic pattern for hybrid wheat breeding. Proceedings of the National Academy of Sciences of the United States of America, 112(51), 15624–15629. 10.1073/pnas.1514547112 26663911 PMC4697414

[tpg220150-bib-0037] Zhao, Y. , Zeng, J. , Fernando, R. , & Reif, J. C. (2013). Genomic prediction of hybrid wheat performance. Crop Science, 53(3), 802–810. 10.2135/cropsci2012.08.0463

