# Supplementary material for: Optimizing the setup of multienvironmental hybrid wheat yield trials for boosting the selection capability
Source: Plant Genome. 2021 Sep 19;14(3):e20150. doi: 10.1002/tpg2.20150 (PMC12893651; doi:10.1002/tpg2.20150)
Supplement: Supplementary file 1 [file TPG2-14-e20150-s001.docx]

# Supplemental material to

Optimizing the setup of multi-environmental hybrid wheat yield trials for boosting the selection capability

Moritz Lell, Jochen Reif, and Yusheng Zhao

Author affiliations:

Moritz Lell (ORCID: 0000-0002-2428-5157), Jochen Reif (ORCID: 0000-0002-6742-265X), and Yusheng Zhao (ORCID: 0000-0001-6783-5182), Leibniz Institute of Plant Genetics and Crop Plant Research (IPK), D-06466, Seeland, Germany. Corresponding author: J. Reif ([reif@ipk-gatersleben.de](mailto:reif@ipk-gatersleben.de))

# CONTENTS

- 5 Supplemental Figures
- 2 Supplemental Tables
- Supplemental Methods

# SUPPLEMENTAL FIGURES

Supplemental Figure S1: Statistics of the Gibbs sampler chains from the GBLUP models to judge sampler convergence. Each plot row shows the statistics for one parameter of the model, that is the intercept (mu) and the variance components. **A:** Effective sample size of the chain, which corrects the number of sampler iterations by the autocorrelation of the chain. **B:** The Geweke diagnostic, a Z score judging whether the beginning and the end of the chain might be drawn from distributions with different expectation values and thus the sampler might not have had a stationary distribution after the end of the burn-in phase.

Supplemental Figure S2: Trace plot of the GBLUP model fit of the ‘S-All’ dataset (see methods part), estimated using Gibbs sampling. Each plot shows the sampler state at each iteration step with regards to one model parameter, that is the intercept (mu) and the variance components (var_*, add = additive, dom = dominance, Env = environment, resid = residual). The dashed line marks the end of the burn-in phase. Note that the Gibbs sampler changes a single parameter value per iteration whereas in this plot each new complete parameter sample is denoted as one iteration step. Therefore, one iteration step corresponds to five Gibbs sampler iterations.

Supplemental Figure S3: Observations of hybrid genotypes in the hybrid wheat data set used in this study. For each hybrid genotype environments where yield observations are available are marked in gray. If no year is printed, it is the same as that of the environment to the left. Number of observations per genotype and environment is indicated by the shade. Abbreviations: ADE = Adenstedt, BOH = Böhnshausen, HAD = Hadmersleben, HHOF = Harzhof, HOH = Hohenheim, SEL = Seligenstadt.

Supplemental Figure S4: Geographical locations of tested locations in Germany (latitude, longitude): Adenstedt (52.3° N, 10.1° E), Böhnshausen (51.9° N, 11.0° E), Hadmersleben (52.0° N, 11.3° E), Harzhof (54.4° N, 9.9° E), Hohenheim (48.7° N, 9.2° E), Seligenstadt (49.9° N, 10.1° E).

# SUPPLEMENTAL METHODS

## Characterization of the environments

The yield phenotypes in each study environment were summarized by calculating the minimum, maximum and mean of the yield observations (Supplemental Table S1). Moreover, we calculated the variance components of the phenotypic variance by fitting the following linear mixed model separately for each environment:

$y_{iklm}=g_{i}+t_{k}+r_{kl}+b_{klm}+\varepsilon_{iklm}$,

(S1)

The observations $y$ are decomposed into the random factors for genotype ($g$), trial ($t$), replication nested in trial ($r$), block nested in replication ($b$) and residual ($\varepsilon$), with$x\sim N\left( 0,I\sigma_{x}^{2} \right)$ for all $x$ in $g,t,r,b,\varepsilon$. The indices are denoted in the same way as for model (1) and following, with the exception of $j$ missing because the model is fitted for each environment separately and it thus would have only one value. The model was fitted using AsReml like model (1). The estimated variance components are shown for each environment in Supplemental Table S2.

For each environment, the repeatability $r^{2}$ was calculated as $r^{2}={\sigma_{g}^{2}}/\left( \sigma_{g}^{2}+\left( {\sigma_{\varepsilon}^{2}}/n \right) \right)$ where $n$ is the average number of observations of the genotypes in the respective environment. The repeatability and its constituent values are listed in Supplemental Table S2 ($n$ is listed as „Avg. obs./gt.“)

Supplemental Table S1: Minimum, maximum and mean yield observed in each of the study environments. The last row summarizes all observations. Details are described in Supplemental Methods.

| Location | Year | min | max | mean |
| --- | --- | --- | --- | --- |
| Böhnshausen | 2012 | 73.3 | 132.7 | 108.6 |
| Hadmersleben | 2012 | 49.1 | 140.8 | 110.7 |
| Harzhof | 2012 | 66.9 | 149.4 | 124.7 |
| Hohenheim | 2012 | 48.2 | 142.7 | 97.2 |
| Seligenstadt | 2012 | 71.1 | 138.4 | 112.8 |
| Adenstedt | 2013 | 54.1 | 127.0 | 93.7 |
| Böhnshausen | 2013 | 64.1 | 131.8 | 102.2 |
| Hadmersleben | 2013 | 77.0 | 134.4 | 111.2 |
| Harzhof | 2013 | 64.9 | 134.0 | 110.9 |
| Hohenheim | 2013 | 33.4 | 137.5 | 98.3 |
| Seligenstadt | 2013 | 67.2 | 115.9 | 92.9 |
| (All) |  | 33.4 | 149.4 | 105.2 |

Supplemental Table S2: Variance components, repeatabilities of the study environments (location times year combinations), as well as heritability. The columns $\sigma_{\cdot}^{2}$ are the variance components from the BLUP model S1 (rows 1-11) or S2 (row 12) that was fitted separately for each environment. The following columns is the average number of observations per genotype (“Avg. obs./gt.”). The last column shows the ratio of genotypic to phenotypic variance. This is equal to the repeatability for all except the last row and the broad-sense heritability for the last row. Details are described in Supplemental Methods.

| Location | Year | $\sigma_{e}$ | $\sigma_{t}$ | $\sigma_{r}$ | $\sigma_{b}$ | $\sigma_{g}$ | $\sigma_{\varepsilon}$ | Avg obs./gt | ${\sigma_{g}}/{\sigma_{\text{P}}}$ |
| --- | --- | --- | --- | --- | --- | --- | --- | --- | --- |
| Böhnshausen | 2012 |  | 3.4 | 0.4 | 2.1 | 30.3 | 22.3 | 1.28 | 0.64 |
| Hadmersleben | 2012 |  | 0.0 | 3.1 | 24.7 | 43.8 | 32.0 | 1.37 | 0.65 |
| Harzhof | 2012 |  | 1.2 | 1.0 | 20.3 | 34.4 | 24.3 | 1.37 | 0.66 |
| Hohenheim | 2012 |  | 0.0 | 21.1 | 24.5 | 35.8 | 33.0 | 1.36 | 0.60 |
| Seligenstadt | 2012 |  | 6.3 | 1.9 | 3.8 | 23.8 | 24.7 | 1.26 | 0.55 |
| Adenstedt | 2013 |  | 22.9 | 2.4 | 49.7 | 23.5 | 21.0 | 1.37 | 0.60 |
| Böhnshausen | 2013 |  | 37.6 | 0.0 | 22.7 | 23.8 | 38.7 | 1.35 | 0.45 |
| Hadmersleben | 2013 |  | 13.3 | 2.7 | 7.0 | 23.0 | 14.1 | 1.34 | 0.69 |
| Harzhof | 2013 |  | 4.9 | 3.6 | 10.1 | 36.0 | 8.2 | 1.37 | 0.86 |
| Hohenheim | 2013 |  | 47.4 | 0.0 | 18.8 | 28.1 | 99.9 | 1.36 | 0.28 |
| Seligenstadt | 2013 |  | 0.0 | 1.8 | 1.0 | 30.7 | 6.0 | 1.37 | 0.87 |
| (All) |  | 89.4 | 11.5 | 4.0 | 17.7 | 17.7 | 41.7 | 13.55 | 0.85 |

## Heritability

The heritability for yield was calculated by fitting the following 1-step linear mixed model, using the observations from all environments:

$y_{ijklm}=g_{i}+e_{j}+t_{jk}+r_{jkl}+b_{jklm}+\varepsilon_{ijklm}$,

(S2)

The model is equal to model S1, with the exception that $g$ is a random effect with $g\sim N\left( 0;I\sigma_{g} \right)$.

The heritability is calculated from the variance components derived from this model as ${\sigma_{g}}/\left( \sigma_{g}+\left( {\sigma_{\varepsilon}}/n \right) \right)$ where $n$ is the average number of observations per genotype in the whole data set. The heritability is listed in Supplemental Table S2 in the column “${\sigma_{g}}/{\sigma_{\text{P}}}$”, last row. The value of $n$ is shown in the same row, column „Avg. obs./gt.“

## Generation of *in silico* scenarios

The scenarios were defined so that all have the same number of observations and 4/11 (38%) of the observations of the data set S-All (Supplemental Figure S5 A).

Each scenario was realized 100 times with a different random sample from the full data set. The sampling was done on the basis of genotypes in environments, not observations, so the realizations contained either all replications of a genotype in an environment (maximum of two observations per genotype) or none. To estimate the influence of the available environments on the results, the scenarios S2–S6 contained a random selection of at most ten of the eleven available environments. Thus, across the realizations, one environment was randomly left out and potential bias by single unusual environments on the result is averaged over.

By the random selection of observations to be included in a scenario realization, the average number of replications of a genotype in one environment stayed constant throughout all scenarios (Supplemental Figure S5 B, C). However, as real-life data was used, some observations failed in the field or did not pass the quality controls and are thus missing in the data set. Therefore, in single realizations there were slight deviations from the norm values.

Supplemental Figure S5: Overview characteristics of environments. The box plots summarize the values attained by the realizations of each scenario. The right axis shows the fraction of the value of S-All. **A:** Total Number of available phenotypic measurements. **B:** Number of hybrid genotypes. **C:** Average replication of a genotype in one environment.
